# Safety of a bivalent, killed, whole-cell oral cholera vaccine in pregnant women in Bangladesh: evidence from a randomized placebo-controlled trial

**DOI:** 10.1186/s12879-019-4006-3

**Published:** 2019-05-15

**Authors:** Ashraful Islam Khan, Mohammad Ali, Julia Lynch, Alamgir Kabir, Jean-Louis Excler, Md. Arifuzzaman Khan, Md. Taufiqul Islam, Afroza Akter, Fahima Chowdhury, Amit Saha, Iqbal Ansary Khan, Sachin N. Desai, Deok Ryun Kim, Nirod Chandra Saha, Ajit P. Singh, John D. Clemens, Firdausi Qadri

**Affiliations:** 1 International Centre for Diarrhoeal Disease Research Bangladesh (icddr,b), 68, Shaheed Tajuddin Ahmed Sarani, Mohakhali Dhaka, 1212 Bangladesh; 2 Johns Hopkins Bloomberg School of Public Health, Baltimore, MD USA; 3 International Vaccine Institute, Seoul, South Korea

**Keywords:** OCV, Cholera, Pregnant women, Safety, Bangladesh

## Abstract

**Background:**

Cholera increases the risk of harmful effects on foetuses. We prospectively followed pregnant women unaware of their pregnancy status who received a study agent in a clinical trial evaluating the association between exposure to an oral cholera vaccine (OCV) and foetal survival.

**Methods:**

Study participants were selected from a randomized placebo-controlled trial conducted in Dhaka, Bangladesh. The vaccination campaign was conducted between January 10 and February 4, 2014. We enrolled women who were exposed to an OCV or placebo during pregnancy (Cohort 1) and women who were pregnant after the vaccination was completed (Cohort 2). Our primary endpoint was pregnancy loss (spontaneous miscarriage or stillbirth), and the secondary endpoints were preterm delivery and low birth weight. We employed a log-binomial regression to calculate the relative risk of having adverse outcomes among OCV recipients compared to that among placebo recipients.

**Result:**

There were 231 OCV and 234 placebo recipients in Cohort 1 and 277 OCV and 299 placebo recipients in Cohort 2. In Cohort 1, the incidence of pregnancy loss was 113/1000 and 115/1000 among OCV and placebo recipients, respectively. The adjusted relative risk for pregnancy loss was 0.97 (95% CI: 0.58–1.61; *p* = 0.91) in Cohort 1. We did not observe any variation in the risk of pregnancy loss between the two cohorts. The risks for preterm delivery and low birth weight were not significantly different between the groups in both cohorts.

**Conclusions:**

Our study provides additional evidence that exposure to an OCV during pregnancy does not increase the risk of pregnancy loss, preterm delivery, or low birth weight, suggesting that pregnant women in cholera-affected regions should not be excluded in a mass vaccination campaign.

**Trial registration:**

The study is registered at (http://clinicaltrials.gov). Identifier: NCT02027207.

## Background

Cholera remains a fatal disease in resource-limited countries, which is caused by *Vibrio cholerae* 01 or 0139 that spreads by water and food contaminated with human faeces. If the infected individuals do not promptly receive adequate treatment, the risk of death can be up to 70% [1]. Cholera during pregnancy may have an adverse effect on pregnancy outcome [2, 3]. The effect of cholera on pregnancy outcome has been observed since the nineteenth century [4]. Much of the published literature on cholera during pregnancies reported pregnancy loss, with the magnitude varying from 2 to 36% [2, 5–9]. A recent study from Haiti reported that pregnant women with severe dehydrating cholera had 9.4 times more risk of delivering a foetal death compared to those with mild dehydrating cholera [10].

A killed whole-cell oral cholera vaccine (OCV) is currently being used as a major tool to control and prevent cholera [11–14]. Theoretically, an OCV should not produce any adverse pregnancy outcomes due to its inability to replicate in the gut and oral route of intake. The World Health Organization (WHO) recommends that pregnant women be included in OCV campaigns because evidence indicates high potential benefit and minimal risks [15]. A mass vaccination campaign conducted in Zanzibar, Tanzania in 2009 with an OCV, Dukoral, did not cause any harmful effects on pregnancies [16]. A retrospective cohort study performed in Guinea in 2013 also showed no evidence of an increased risk of pregnancy loss after receiving Shanchol [17]. Recently, a retrospective study conducted in Bangladesh and a prospective study conducted in Malawi did not find any harmful effects of an OCV in pregnant women [18, 19]. However, the package insert for OCVs still recommends caution for use during pregnancy due to limited safety data in this population group. Since most of the studies were carried out in African countries during cholera outbreaks, more evidence is needed from cholera endemic areas, in particular, from parts of the world known to have a high prevalence of cholera.

We conducted an individually randomized, placebo-controlled trial of a single-dose regimen of the OCV Shanchol. As per the package insert for Shanchol, individuals 1 year or older and non-pregnant were invited to participate in the trial. All married women of childbearing age (13–49 years) were verbally screened for pregnancy before dosing. Since pregnancy in the first trimester may not be visible or some women may experience irregular menstruation, several pregnant women took a study agent unaware of their pregnancy status at the time of dosing. In this study, we evaluated the effect of vaccination on pregnant women who were unaware of their pregnancy status and who inadvertently received an OCV. The main goal of this study was to evaluate the effect of vaccination on pregnant women who received the OCV without knowing their pregnancy status.

## Methods

### Study population

The clinical trial was conducted in an urban setting in Dhaka, Bangladesh, where cholera is highly endemic [20]. A total of 204,700 residents in the area underwent randomization and received a single dose of OCV. A baseline census was conducted during November 12, 2012 and January 29, 2013 to enumerate the regular residents in the study area. This census was updated immediately before the initiation of vaccine administration in 2014. Details of the study area, randomization, and the study procedure were published elsewhere [20]. In this analysis, we considered only the women of childbearing age who had received study agents.

### Mass vaccination campaign

The mass vaccination campaign was conducted from 10 January 2014 to 4 February 2014 with technical assistance from the Expanded Programme on Immunization (EPI) of the Ministry of Health and Social Welfare, City Corporation, International Vaccine Institute (IVI) and other stakeholders. The study agents were offered to the eligible subjects in an individually randomized fashion. Each dose of vaccine or placebo was 1.5 ml in volume. Placebo vials contained only the inert constituents starch and xanthan gum. The study agents were dispensed in liquid form in identical vials in a double-blind manner. The details of the composition of the OCV were described previously [12]. Since it was not feasible to perform a pregnancy test during the vaccination campaign, the pregnancy status and date of the last menstrual period (LMP) were inquired verbally for all married women of childbearing age (13–49 years) prior to vaccination. If the date of the LMP was more than 4 weeks, irregular periods, unknown, or uncertain, we considered them ineligible for vaccination.

### Enrolment of study subjects and follow-up

During the screening visit, between the 22nd of April and 10th of July 2014 (approximately 3–5 months after the vaccination campaign was completed), trained female field staff screened the pregnancy status of all women of childbearing age receiving the study agents. After obtaining verbal consent, a structured questionnaire was used in the interviews. A household was visited a maximum of 3 times if a potential woman was found absent. If a woman was not sure about her pregnancy or could not confirm her pregnancy status during the visit, she was re-visited 1 month later. After confirmation of the pregnancy status of the potential women, the field staff notified this result to the study physicians with the list of pregnant women for enrolment in the study and further follow-up. Scheduled monthly phone calls were made to each participant to inquire about the pregnancy status until 6 months of her gestational age. If a pregnancy outcome was notified during the screening visit, this report was considered a retrospective follow-up. The study physicians interviewed the woman about the outcome of her pregnancy after obtaining written informed consent. If women were found pregnant (whose outcome had not occurred), they were termed prospective follow-up.

From 6 months of gestational age, each participant was followed until miscarriage, stillbirth or end of pregnancy. The study physicians conducted monthly home visits for the follow-up interviews. Informed written consent was obtained from the participants prior to the initial interview. In the case of minor participants, consent was obtained from the parents or guardians on the same consent form. Detailed information on pregnancy, such as obstetrical history and clinical history, including anthropometrical measurement (height, weight and mid-upper arm circumference), was collected from the participants. Physicians’ contact numbers were given to the participants for notifying them if the subjects would change their current address or if any outcome occurred before the next visit. Weekly phone calls were made after 8 months of gestational age. A final home visit was made within 1 week after pregnancy outcome when anthropometrical measurements (height, weight) of the live newborn were taken. Moreover, adverse pregnancy outcomes (pregnancy loss or any congenital anomalies) were recorded immediately after the event.

### Data analysis

In the primary analysis, we included women who were pregnant during vaccination whose foetuses were exposed to a study agent (Cohort 1), and in the secondary analysis, we included women who became pregnant just after vaccination whose foetuses were not exposed to a study agent (Cohort 2). We compared the characteristics of the vaccine and placebo recipients using the chi-squared test (or Fisher’s exact test for sparse data) and Student’s t-test (or Mann-Whitney’s test for non-normal data) for binary and continuous variables, respectively, for each cohort. The primary endpoint was pregnancy loss (spontaneous miscarriage or stillbirth). We defined spontaneous miscarriage as termination of a pregnancy without a known external cause prior to 28 weeks of gestation [21], and stillbirth was defined as delivery of a dead foetus at 28 weeks or later [22]. The secondary endpoints were preterm delivery (defined by pregnancy ending with a live birth < 37 weeks of gestational age) and low birth weight (birth weight < 2500 g). We also performed a supplementary analysis assessing a potential effect of an OCV on induced or accidental abortion.

We used a log-binomial model and calculated relative risk (RR) after adjusting for potential confounders. The confounders were selected from the bivariate analysis, which appeared imbalanced between vaccine and placebo recipients at *p* < 0.20 and following the rule of 10 events per covariate to maximize the coverage of the confidence interval of the estimate from a regression model [23]. The strength of the relationship of one over the other covariates was used in selecting the covariates when following the rule of 10 events per covariate. All *p*-values and 95% confidence intervals were interpreted in a two-tailed fashion. All analysis was performed in R version 3.2.3 (Vienna, Austria, 2016).

## Result

**Fig. 1:**
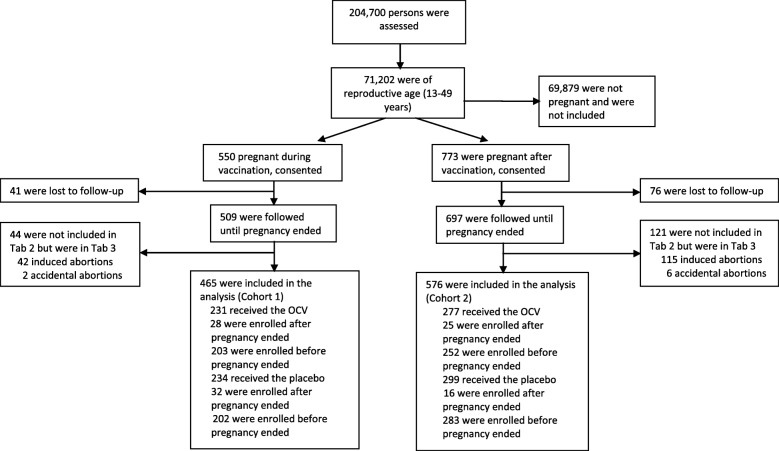
Assembling the study population for analysis

A total of 204,700 individuals participated in the clinical trial, of whom 71,202 were women of reproductive age (13–49 years). During the screening visit, we identified 1323 pregnancies. Among them, we could recruit 550 pregnant women in Cohort 1 and 773 in Cohort 2. We lost to follow-up 41 women in Cohort 1 and 76 women in Cohort 2 between vaccination and screening visits. Due to induced or accidental abortion, we excluded 44 women in Cohort 1 and 121 women in Cohort 2. Finally, we analysed 465 women in Cohort 1 and 576 women in Cohort 2. We prospectively followed 405 (87%) of 465 women in Cohort 1 and 535 (93%) of 576 women in Cohort 2, and the rest were followed retrospectively (Fig. 1).

There were 231 OCV recipients and 234 placebo recipients in Cohort 1 and 277 OCV and 299 placebo recipients in Cohort 2. Socio-demographic, nutritional, and obstetric characteristics of the pregnant women were balanced between OCV recipients and placebo recipients except for having diarrhoea in the past 6 months prior to registration in the baseline census among women in Cohort 2 (Table 1). In Cohort 1, there were 26 adverse pregnancy outcomes in the OCV group and 27 in the placebo group. In this cohort, the incidence rate of an adverse pregnancy outcome was 112.6 per 1000 pregnancies among OCV recipients and 115.4 per 1000 pregnancies among placebo recipients (Table 2). Incidence of preterm delivery was 33.8, and of low birth weight was 97.6 per 1000 live births among OCV recipients, and 58.0 and 111.1, respectively, among the placebo recipients. The risk of having an adverse pregnancy outcome among women exposed to the OCV was 0.97 (95% CI: 0.58–1.61, *p* = 0.907) compared to that of women exposed to placebo. No significant risk was observed for having a preterm delivery or low birth weight among women exposed to the OCV compared to those of the placebo recipients (Table 2).

**Table 1:** Socioeconomic and obstetric characteristics of the OCV recipients and placebo recipients among pregnant women

Characteristics	Pregnant during vaccination (Cohort 1)			Pregnant after vaccination (Cohort 2)		
	OCV recipients (*n* = 231)	Placebo recipients (*n* = 234)	p-value	OCV recipients (*n* = 277)	Placebo recipients (*n* = 299)	*p*-value
Median gestational age at screening visit/recruitment– (IQR; weeks)	22 (19, 25)	21 (19, 25.5)	0.541	12 (9, 15)	12 (9, 16)	0.751
Median gestational age at vaccination – (IQR; weeks)	4 (1.79, 7.71)	4.43 (2.14, 8.57)	0.407	–	–	–
Median time of vaccination prior to pregnancy -(IQR; weeks)	–	–	–	4.71 (2.14, 8.14)	5 (2.43, 8.29)	0.408
Exposed to the OCV in the 1st trimester– no. (%)	198 (85.71)	194 (82.91)	0.481			
Mean age (SD; year)	26.27 (5.70)	26.19 (5.70)	0.876	24.92 (5.10)	24.81 (5.26)	0.785
Illiterate– no. (%)	53 (22.94)	49 (20.94)	0.682	39 (14.08)	54 (18.06)	0.236
Diarrhoea within past 6 months prior to baseline census-- no. (%)	24 (10.39)	28 (11.97)	0.695	31 (11.19)	16 (5.35)	0.016
Live in own house – no. (%)	37 (16.02)	34 (14.53)	0.751	59 (21.30)	62 (20.74)	0.949
Live in a household with family size ≤4 – no. (%)	112 (48.48)	108 (46.15)	0.681	112 (40.43)	106 (35.45)	0.252
Live in a household using own tap as the source of drinking water-– no. (%)	15 (6.49)	13 (5.56)	0.818	18 (6.50)	31 (10.37)	0.130
Live in a household with treated drinking water-– no. (%)	127 (54.98)	133 (56.84)	0.756	177 (64.13)	189 (63.21)	0.887
Live in a household using sanitary toilet– no. (%)	156 (67.53)	167 (71.37)	0.425	214 (77.26)	219 (73.24)	0.309
Median time living in the household (IQR; months)	20 (7, 48)	15 (6, 38.5)	0.369	24 (10, 48)	24 (6, 48)	0.683
Live in a household with concrete roof – no. (%)	37 (16.02)	31 (13.25)	0.475	48 (17.33)	70 (23.41)	0.088
Live in a household with concrete wall – no. (%)	161 (69.70)	160 (68.38)	0.835	205 (74.01)	212 (70.90)	0.460
Live in a household using shared kitchen– no. (%)	197 (85.28)	202 (86.32)	0.850	232 (83.75)	253 (84.62)	0.866
Live in a household using shared toilet– no. (%)	206 (89.18)	216 (92.31)	0.315	242 (87.36)	258 (86.29)	0.796
Live in a household with only one room– no. (%)	54 (23.38)	54 (23.08)	1.000	82 (29.60)	77 (25.75)	0.347
Obstetric history						
Had any ANC visit^a^– no. (%)	191 (82.68)	189 (80.77)	0.679	246 (88.81)	267 (89.30)	0.957
Had ankle swelling– no. (%)	11 (4.76)	18 (7.69)	0.265	15 (5.42)	18 (6.02)	0.894
Had gestational diabetes^b^– no. (%)	4 (1.73)	2 (0.85)	0.448	1 (0.36)	4 (1.34)	0.375
Had gestational hypertension^c^– no. (%)	1 (0.43)	4 (1.71)	0.372	4 (1.44)	8 (2.68)	0.387
Had eclampsia^`d^– no. (%)	1 (0.43)	0 (0.00)	0.497	3 (1.08)	1 (0.33)	0.356
Had history of stress^e^– no. (%)	26 (11.26)	23 (9.83)	0.726	29 (10.47)	38 (12.71)	0.479
Had previous bad obstetrical history^f^– no. (%)	78 (33.77)	76 (32.48)	0.844	102 (36.82)	115 (38.46)	0.749
Had any chronic disease^7^– no. (%)	9 (3.90)	6 (2.56)	0.582	11 (3.97)	12 (4.01)	1.000
Mid-upper arm circumference ≤ 21.5 cm– no. (%)	11 (6.36)	11 (6.79)	1.000	11 (4.76)	13 (5.20)	0.991

^a^Antenatal care is the routine health control of presumed healthy prengant women without symptoms (screening) to diagnose diseases or complicating obstetric conditions without symptoms and to provide information about lifestyle, pregnancy and delivery

^b^Gestational diabetes is a high blood sugar that develops during pregnancy and usually disappears after giving birth

^c^Gestational hypertension is new hypertension presenting after 20 weeks without significant proteinuria

^d^A convulsive state; especially: an attack of convulsions during pregnancy or childbirth

^e^In a medical or biological context, stress is a physical, mental, or emotional factor that causes bodily or mental tension

^f^The term ‘Bad Obstetric History or BOH’ is applied to mothers in whom a previous poor pregnancy outcome is likely to have a bearing on the prognosis of her present pregnancy

^7^The term chronic disease applies to a group of diseases that tend to be long lasting and have persistent effects

**Table 2:** Relative risk (RR) of having an adverse pregnancy outcome among OCV recipients compared to that of placebo recipients

Pregnancy outcome	OCV recipients		Placebo recipients		Crude RR (95% CI, *p*-value)	Adj. RR (95% CI, *p*-value)
	Cases/ Population	Rate/1000	Cases/ Population	Rate/1000		
*Pregnant during vaccination (Cohort 1)*						
Adverse pregnancy outcome	26/231	112.6	27/234	115.4	0.98 (0.58–1.62, 0.923)	0.97^a^ (0.58–1.61, 0.907)
Miscarriage	20/231	86.6	22/234	94.0	0.92 (0.51–1.64, 0.780)	0.91^a^ (0.51–1.63, 0.752)
Stillbirth	6/211	28.4	5/212	23.6	1.21 (0.37–4.13, 0.754)	--^d^
Preterm delivery	7/207	33.8	12/207	58.0	0.59 (0.22–1.43, 0.255)	0.58^a^ (0.22–1.40, 0.237)
Low birth weight	20/205	97.6	23/207	111.1	0.88 (0.49–1.55, 0.653)	0.88^a^ (0.49–1.55, 0.656)
*Pregnant after vaccination (Cohort 2)*						
Adverse pregnancy outcome	39/277	140.8	39/299	130.4	1.08 (0.71–1.63, 0.717)	1.02^b^ (0.67–1.55, 0.930)
Miscarriage	36/277	130.0	32/299	107.0	1.21 (0.78–1.91, 0.395)	1.15^b^ (0.73–1.81, 0.555)
Still birth	3/241	12.4	7/267	26.2	0.47 (0.10–1.69, 0.276)	-^d^
Preterm delivery	12/238	50.4	21/260	80.8	0.62 (0.30–1.22, 0.179)	-^d^
Low birth weight	19/238	79.8	26/260	100.0	0.80 (0.45–1.40, 0.434)	0.82^c^ (0.46–1.43, 0.477)

^a^ Adjusted for gestational age at vaccination

^b^ Adjusted for time of vaccination prior to pregnancy (included by choice due to biological plausibility), diarrhoea in past 6 months and roof material of household

^c^ Adjusted for diarrhoea in the past 6 months

^d^ Number of cases was not sufficient to perform a multivariable model

In Cohort 2, there were 39 adverse pregnancy outcomes in each of the study groups. The incidence rate of an adverse pregnancy outcome in this cohort was 140.8 per 1000 pregnancies among OCV recipients and 130.4 per 1000 pregnancies among placebo recipients (Table 2). The rate of preterm delivery was 50.4 and of low birth weight was 79.8 per 1000 live births in OCV recipients and 80.8 and 100.0, respectively, in placebo recipients. There were a total of 26 adverse pregnancy outcomes, including 20 miscarriages and 6 stillbirths in Cohort 1. In Cohort 2, there were 32 miscarriages, and 7 still births occurred. There was also no statistically significant difference in the risk of pregnancy loss among OCV recipients in Cohort 2 compared to that among placebo recipients of this cohort, and the rates of preterm delivery and low birth weight were similar between the two groups of this cohort (Table 2).

Table 3 presents the effect of an OCV on induced or accidental abortion. Among women pregnant during vaccination (*n* = 509), the rates of induced or accidental abortion were 83.3 and 89.5 per 1000 pregnancies among OCV and placebo recipients, respectively. The risk of induced or accidental abortion was not significant [RR = 0.93, 95% CI: 0.52–1.64]. Among women pregnant after vaccination (*n* = 697), the rates of induced or accidental abortion were 178.0 and 169.4 per 1000 pregnancies among OCV and placebo recipients, respectively. We found no risk due to the OCV on induced or accidental abortion [RR = 1.05, 95% CI: 0.76–1.45].

**Table 3:** Relative risk of having an abortion (induced or accidental) among OCV recipients compared to that of placebo recipients

Pregnancy outcome	OCV recipients		Placebo recipients		Crude RR (95% CI, *p*-value)	
	Cases/ Population	Rate/1000	Cases/ Population	Rate/1000		
*Pregnant during vaccination*						
Abortion	21/252	83.33	23/257	89.5	0.93 (0.52–1.64, 0.805)	
*Pregnant after vaccination*						
Abortion	60/337	178.0	61/360	169.4	1.05 (0.76–1.45, 0.765)	

## Discussion

The results of our study suggest that administration of an OCV during pregnancy was not associated with adverse pregnancy outcomes. Although not statistically significant, the rate of adverse pregnancy outcomes among the OCV recipients was slightly lower compared to that among the placebo recipients. There were a total of 26 adverse pregnancy outcomes, including 20 miscarriages and 6 stillbirths in Cohort 1. In Cohort 2, there were 32 miscarriages, and 7 still births occurred. Previously reported studies in pregnant women receiving an OCV have observed a non-significant increase in adverse pregnancy outcomes among women receiving the OCV [16, 17]. Unlike those studies, our samples were taken from an individually randomized trial population; therefore, it is less likely that our results are biased by sample selection. Additionally, the different adverse pregnancy outcome rates (miscarriage and stillbirth) were also similar between OCV and placebo recipients, suggesting that an OCV (killed, non-replicating, oral administration) does not have any impact on foetus survival. Our results reinforce previous findings that OCVs do not have any negative impact on preterm delivery, low birth weight or congenital anomalies. Based on several studies, the WHO recommends that an OCV be given to women who are pregnant [18, 19, 24]. Altogether, these data suggest that an OCV can be administered during pregnancy.

In our study, we observed that the miscarriage and stillbirth rates were comparable in both cohorts. In a survey conducted between during 1982 and 2002 in a rural area approximately 55 km southeast of Dhaka, we observed 53 miscarriages per 1000 pregnancies and 30 stillbirths per 1000 births [21]. In another study conducted in northwest Bangladesh during 2001 and 2007, 88 miscarriages per 1000 pregnancies were observed, using a miscarriage definition of < 24 weeks of gestation [25]. According to a recent report, there are 19 stillbirths per 1000 births in Bangladesh [22]. These data suggest that the rate of adverse pregnancy outcomes differs by the setting and by the method of detection in Bangladesh and may vary over time. In addition, it remains difficult to categorize abortion as induced or spontaneous because of legal and moral loopholes. Under the penal code of 1860, induced abortion is illegal in Bangladesh except when performed to save a women’s life.

Cholera causes maternal dehydration during pregnancy, which leads to critical hypovolemia that compromises placental and foetal perfusion and eventually leads to foetal death [24]. As a result, miscarriage or premature delivery may occur. Moreover, different studies have reported that pregnancy may be associated with poor outcomes in cases of delayed rehydration therapy [2, 10]. Women who reported having had cholera while they were pregnant were at 6-fold higher risk of miscarriage and 3-fold higher risk of having a stillborn child than women who did not have cholera [17]. Data from Senegal demonstrated that pregnant women took four times longer to reach a health facility for cholera treatment than the general population [9]. Therefore, preventive measures such as vaccination could be a better choice to prevent cholera during pregnancy, which will reduce the risk of pregnancy loss.

The unique strength of our study is that we drew our samples from a double-blinded randomized study, suggesting that the results of our study are free from sampling bias. We initiated screening immediately after vaccination and could capture many participants (84%) receiving the OCV in their 1st trimester. We also followed the participants prospectively, which minimizes recall bias. The follow-ups were made by trained physicians to correctly classify pregnancy outcomes.

However, our study had some limitations. First, our study was conducted in an endemic setting where several subjects may have developed some preexisting immunity. Although there is no clear knowledge of the effect of preexisting immunity of OCV recipients on foetal survival, our results may not apply in non-endemic settings. Evidence from the studies conducted in the past is not different from our results, suggesting that the pregnancy outcomes among OCV recipients are not influenced by the type of setting. Second, although our aim was to conduct a prospective study, 10% of pregnancies were already terminated at the time of recruitment. Since a large proportion of the women were prospectively followed and the number of women retrospectively followed was similar in both groups, we do not think that the small proportion of the retrospectively followed women affected the analysis. Third, we tested only a single dose, whereas the recommended dose of an OCV is a double dose. Another potential limitation is that we did not find any congenital anomalies of the infants. However, the expected prevalence of congenital anomalies among newborns is approximately 2% [26], and the probability of not detecting such defects by chance is 8% (calculated from a binomial distribution with 500 trials).

Due to insufficient safety data, cholera vaccination campaigns frequently exclude pregnant women based on information in package inserts of the OCV. However, according to the WHO, individuals who are at risk of cholera and for whom vaccines are not contraindicated should be targeted by the OCV. Many inactivated vaccines are already given to pregnant women, including tetanus, diphtheria, hepatitis B, flu, and pneumococcal vaccines [27], whereas the OCV used in this study is also an inactivated killed vaccine given orally and acts locally in the intestine [28].

## Conclusion

Women in an endemic or outbreak setting are at risk of cholera, and contraction of the disease during pregnancy can cause miscarriages or stillbirths [3]. Our study confirms that there was no risk of foetal loss due to receiving an OCV during pregnancy. Therefore, when the risk of cholera infection is high, an OCV should be offered to pregnant women since they are particularly at a high risk of losing their foetus if they become infected with cholera.

## Abbreviations

EPI: Expanded Programme on Immunization
IVI: International Vaccine Institute
LMP: last menstrual period
OCV: Oral cholera vaccine
RR: relative risk
WHO: World Health Organization
